# Direct navigated laser photocoagulation as primary treatment for retinal arterial macroaneurysms

**DOI:** 10.1186/s40942-018-0133-z

**Published:** 2018-08-22

**Authors:** Dmitrii S. Maltsev, Alexei N. Kulikov, Bhushan Uplanchiwar, Luiz H. Lima, Jay Chhablani

**Affiliations:** 1 Department of Ophthalmology, Military Medical Academy, 5 Klinicheskaya St, St. Petersburg, Russia 194044; 2 Smt. Kanuri Santhamma Retina Vitreous Centre, L.V. Prasad Eye Institute, Kallam Anji Reddy Campus, Banjara Hills, Hyderabad, 500 034 India; 3 Department of Ophthalmology, Federal University of São Paulo, São Paulo, Brazil

**Keywords:** Retinal arteriolar microaneurysm, Laser photocoagulation, Navigated laser, Optical coherence tomography, Anti-VEGF therapy

## Abstract

**Objectives:**

To compare the efficacy and safety of conventional and navigated laser photocoagulation as the primary treatment option for retinal arteriolar macroaneurysm (RAM).

**Methods:**

Eleven (9 male and 2 females, mean age 65.1 ± 12.1 years) and 17 (13 male and 4 females, mean age 66.2 ± 8.9 years) patients were included in conventional laser photocoagulation (CLP) and navigated laser photocoagulation (NLP) groups, respectively. The primary outcome measures were LogMAR best-corrected visual acuity (BCVA) and central retinal thickness at the end of the follow-up. The secondary outcome measure was total laser energy applied during the procedure.

**Results:**

At the end of the mean follow-up of 11.4 ± 4.0 months, baseline LogMAR BCVA increased significantly from 0.65 ± 0.14 to 0.26 ± 0.12 (p < 0.001) in CLP group and from 0.57 ± 0.33 to 0.29 ± 0.34 (p < 0.001) in NLP group. Central retinal thickness decreased significantly from 514.5 ± 53.2 µm to 295.3 ± 11.3 µm (p < 0.001) and from 494.0 ± 111.2 µm to 285.8 ± 51.4 µm (p < 0.001) in CLP and NLP group, respectively. Total laser energy and number of laser burns applied per procedure in NLP group was statistically significantly lower than in CLP group (0.28 ± 0.13 J vs 0.59 ± 0.06 J, p < 0.001 and 28.5 ± 14.2 burns vs 48.9 ± 5.1 burns, respectively, p < 0.001). No adverse events related to laser treatment was noted in study groups during the follow-up.

**Conclusion:**

This study demonstrated superiority of navigated laser photocoagulation compared to conventional laser photocoagulation in primary treatment of RAM which results from similar efficacy and safety of both techniques with lower mean total laser energy and number of laser burns required for navigated laser photocoagulation.

Retinal arterial macroaneurysm (RAM) is a local dilatation of the arterial retinal vessel associated with a potential to functional decompensation and disruption of the arterial wall. Risk factors associated with RAM include local (branch retinal vein occlusion (BRVO)) and systemic (arterial hypertension) conditions [1, 2]. Although, independently of the cause, the RAM could be asymptomatic, it has associated risk of: (1) hemorrhagic complications (preretinal, intraretinal, and subretinal hemorrhage) and/or (2) exudation (leading to accumulation of subretinal or intraretinal fluid) involving the center of the macula [3].

Since RAM, if not secondary to retinal vascular disorders (such as BRVO), can resolve spontaneously (or due to compensation of systemic risk factors, e.g., arterial hypertension), observation is suggested as a primary option for RAM management. Among interventional treatment options for symptomatic RAM, laser photocoagulation [4] and anti-VEGF therapy [5] are the most common. Despite its proven efficacy and relative simplicity, conventional laser photocoagulation is not without the possible complications associated with laser therapy in general, including secondary choroidal neovascularization [6] or central scotoma associated with chorioretinal scar [7]. Moreover, the collateral damage of retinal tissue adjacent to the RAM is an almost unavoidable part of conventional laser photocoagulation even if laser burns targeted exclusively to the RAM surface.

In order to reduce the potential harmful effects of laser photocoagulation and risks of adverse events associated with laser treatment, Parodi M and coauthors suggested micropulse laser therapy of the RAM, which they demonstrated has efficacy equal to conventional laser without the attendant collateral retinal damage [8].

Another tissue-sparing approach to laser therapy is the navigated laser photocoagulation. The currently commercially available navigated laser system (NAVILAS; OD-OS GmbH, Teltow, Germany) incorporates a digital fundus camera (provides color fundus imaging, infrared imaging, and fluorescein angiography) and a diode-pumped solid-state (532 or 577 nm) laser. The system allows preplanning for laser procedures using a wide spectrum of images (optical coherence tomography (OCT) [9, 10], intravenous contrast angiography [10, 11], scanning laser ophthalmoscopy [12]) which can be superimposed on the baseline image followed by placing of laser spot marks. During the treatment procedure the retinal tracking system stabilizes the position of laser spot minimizing icorrect application of laser burns [13, 14]. Therefore, navigated laser photocoagulation demonstrated improved precision compared to conventional laser for focal laser photocoagulation of microaneurysms in diabetic macular edema [15]. Nevertheless, the application of navigated laser photocoagulation in the treatment of primary RAM has not been described.

This study aims to compare the efficacy and safety of conventional and navigated laser photocoagulation in the treatment of primary RAM as well as appropriate laser energy required to treat RAM.

## Methods

### Study population

This was a three-center retrospective study, which included symptomatic patients with RAM, confirmed by fluorescein angiography (FA), who underwent direct laser photocoagulation. Exclusion criteria were: absence of, or reported improvement in patient’s visual complaints; best-corrected visual acuity (BCVA) of 20/20 or better; RAM located outside the retinal vascular arcades; opacification of optical media impeding laser procedure; any previous treatment for RAM; optical coherence tomography (OCT) signal strength 5/10 or lower. Patients were assigned to either (1) conventional laser photocoagulation (CLP) or (2) navigated laser photocoagulation (NLP) group. Out of three study centers at two centers patients received only navigated laser while at one study center patients received only conventional laser. The selection of the treatment method was based on preferable practice and the equipment available.

### Baseline examination

All patients received a comprehensive ophthalmic examination. BCVA was measured by the Snellen chart and converted to LogMAR for statistical analysis. FA was performed using scanning laser ophthalmoscope F-10 (NIDEK, Gamagori, Japan) or Spectralis (Heidelberg Engineering, Carlsbad, CA, USA) in accordance with standard procedure including intravenous infusion of 5 ml 10% fluorescein sodium solution.

Prior to laser treatment, all patients were observed for at least 1 month to exclude spontaneous improvement in RAM status and associated retinal exudation. Laser treatment was only indicated if the patient demonstrated visual deterioration and retinal exudation involving the center of the macula, defined as central retinal thickness (CRT) more than 250 µm.

### Optical coherence tomography

The OCT images were taken using Copernicus REVO (Optopol, Zawiercie, Poland), RTVue-100 (Optovue, Fremont, CA, USA) or Spectralis (Heidelberg Engineering, Heidelberg, Germany). For patients examined with Copernicus REVO was obtained 3D-Retina scan (7 mm × 7 mm, 163 B-scans each of 320 A-scans), centred on the center of the macula and line scan (7 mm, 50 B-scans each of 1024 A-scans) crossing the RAM. An OCT retinal thickness map (Enhanced Macular Map 5 (EMM5) protocol) and background SLO-type image (7 mm × 7 mm) were acquired on the RTVue–100. Additionally, with the RTVue–100, a line scan (16 B-scans each of 1024 A-scans averaged in a single scan) crossing the RAM was used to obtain a cross-sectional image of the RAM. For the patients examined with Spectralis, a central scan (19 line scans within 6 mm × 6 mm area each of 9 B-scans each of 1024 A-scans) was used, centered on the center of the macula and the RAM. OCT was performed as a part of the baseline examination, before the laser procedure (after one month of observation), and monthly during the follow-up. Central retinal thickness, the distance between the RAM and the center of the macula, the retinal thickness at the RAM, accumulation of intraretinal or subretinal fluid, preretinal or intraretinal or subretinal hemorrhage, and presence of hard exudates were evaluated in each patient before laser treatment and at the end of follow-up.

### Laser photocoagulation

Laser photocoagulation procedure in NLP group was performed with NAVILAS 532 or NAVILAS 577 laser system and in CLP group using single-spot laser OcuLight GLx 532 nm (Iridex, USA). In both groups, the laser was applied directly to the RAM surface, thus avoiding photocoagulation of the surrounding retina. All procedures were performed with a single session by experienced specialists. Because of retrospective nature of this study there was no standard laser settings for all study centers. In each case the laser spot size (ranged from 100 to 300 µm) and the pulse duration (ranged from 100 to 280 ms) were adjusted based on personal experience of each specialist.

Treatment endpoint was defined as blanching of the RAM, laser power being gradually increased up to the necessary level during the procedure. After sufficient laser power was achieved, the RAM was treated in a confluent manner placing one burn only on each site of the RAM surface.

### Outcome measures

Primary outcome measures were BCVA and CRT at the end of the follow-up. Minimum follow-up was set at 6 months. The secondary outcome measure was the total laser energy applied per RAM, which was calculated as [Total laser energy = burns number × pulse power × pulse duration].

### Statistics

Statistical analysis was performed by means of MedCalc 18.4.1 (MedCalc Software, Ostend, Belgium). Continuous variables are presented as the mean ± SD. To compare BCVA, CRT, and the total laser energy between the study group, independent samples t-test was used. Fisher’s exact test was performed to compare categorical variables. Wilcoxon test was applied to evaluate the statistical significance of the changes in BCVA and CRT after laser photocoagulation. For all statistical methods used, p < 0.05 was considered statistically significant.

## Results

A total of 28 eyes of 28 patients with RAM were included in this study. Eleven (9 male and 2 females, mean age 65.1 ± 12.1 years) and 17 (13 male and 4 females, mean age 66.2 ± 8.9 years) patients were included in CLP and NLP groups, respectively. The mean age in CLP and NLP groups was 65.7 ± 12.1 and 66.2 ± 8.7 years (p = 0.45), respectively. At the baseline, neither BCVA (0.65 ± 0.14 and 0.57 ± 0.33 (p = 0.16) in CLP and NLP groups, respectively) nor CRT (514.5 ± 53.2 µm and 494.0 ± 111.2 µm (p = 0.35) in CLP and NLP groups, respectively) was statistically significantly different between the study groups. The distribution the OCT findings (intraretinal or subretinal fluid, preretinal or intraretinal or subretinal hemorrhage, and hard exudates) was not significantly different between the study groups at the baseline and the end of the follow-up. The distance from the RAM to the center of the macula was 782.2 ± 172.1 µm and 849.6 ± 498.7 µm (p = 0.051), in CLP and NLP groups, respectively. In both groups RAMs were equally distributed between the upper and lower temporal vascular arcades (6 and 5 in CLP group vs 10 and 7 in NLP group, respectively). Additionally, there was no statistically significant difference between the study groups in the retinal thickness at the RAM 554.3 ± 46.0 µm and 534.5 ± 107.5 µm (p = 0.94).

**Fig. 1:**
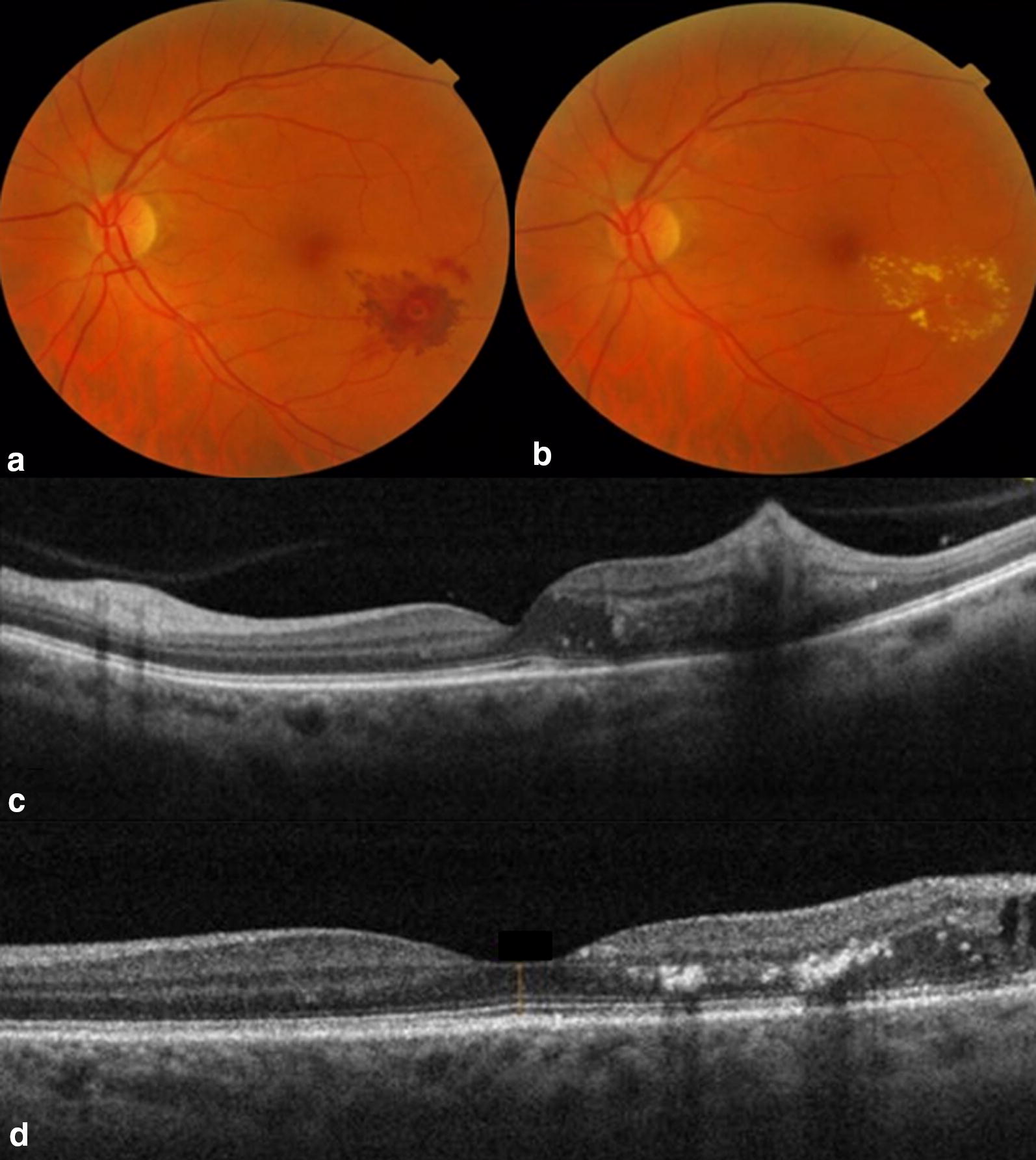
Color fundus photographs demonstrating retinal arteriolar microaneurysm (RAM) appearance in the left eye of 59-years old male before (**a**) and three months after (**b**) the navigated laser photocoagulation procedure. Note significant whitening of the retina surrounding the RAM and local preretinal hemorrhage. Cross-sectional optical coherence tomography scans before navigated laser photocoagulation procedure (**c**) and three months after the procedure (**d**) demonstrate significant improvement in retinal exudation. Best corrected visual acuity increased from 20/63 to 20/32

At the end of the follow-up, the mean BCVA in CLP and NLP groups statistically significantly increased to 0.26 ± 0.12 (p < 0.001) and to 0.29 ± 0.34 (p < 0.001), respectively, however, without difference between the groups (p = 0.54). At the end of the follow-up, the CRT in CLP and NLP groups statistically significantly decreased to 295.3 ± 11.3 µm (p < 0.001) and to 285.8 ± 51.4 (p < 0.001) and was not significantly different between the two groups (p = 0.61). No treatment-associated adverse effects were met during the follow-up period of 11.4 ± 4.0 months (Fig. 1).

**Fig. 2:**
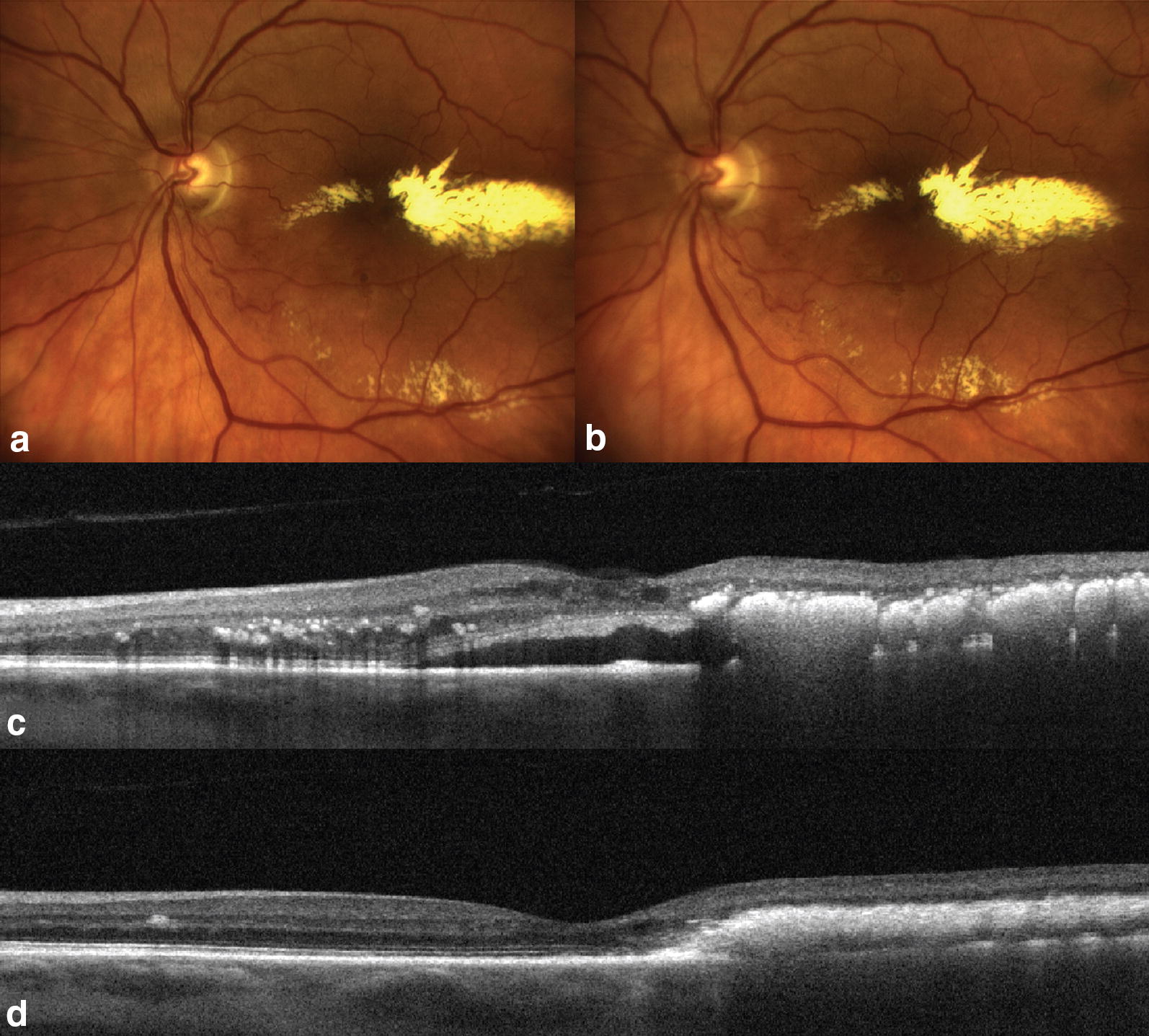
Color fundus photographs demonstrating retinal arteriolar microaneurysm (RAM) appearance in the left eye of 79-years old female before (**a**) and immediately after (**b**) navigated laser photocoagulation procedure. Note subtle whitening of the RAM without changes in the surrounding retina. Cross-sectional optical coherence tomography scans before navigated laser photocoagulation procedure (**c**) and 2 months after the procedure (**d**) demonstrate significant improvement in retinal exudation. Best corrected visual acuity increased from 20/100 to 20/40

Total laser energy used per procedure in CLP group was statistically significantly higher than that in NLP group, 0.59 ± 0.06 versus 0.28 ± 0.13 J, respectively (p < 0.001) (Fig. 2). Laser settings including pulse duration (100.0 ± 0.0 ms and 124.7 ± 57.3 ms (p = 0.20), in CLP and NLP groups, respectively) and burn size (150.0 ± 0.0 µm and 110.0 ± 80.0 µm (p = 0.16) in CLP and NLP groups, respectively) were not statistically significantly different between the study groups. However, the mean number of laser burns applied was significantly higher in CLP compared to NLP group (48.9 ± 5.1 and 28.5 ± 14.2 burns, respectively, p < 0.001).

## Discussion

This study demonstrated that the navigated approach in direct laser photocoagulation as a primary treatment for RAM has similar efficacy and safety compared to conventional photocoagulation. At the same time, navigated laser treatment requires less total laser energy per procedure. Since the study groups did not demonstrate difference in laser burn size and pulse duration, only a decreased number of laser burns are applied to RAM is responsible for the reduction of total laser energy required per procedure. Hence, the potential advantage of the navigated approach in RAM treatment is reduced collateral damage of the retina and retinal pigment epithelium due to the improved accuracy of laser burn application as a result of eye movement compensation by the laser system tracking [13]. Thus, along with similar efficacy and safety, improved accuracy lends superiority to navigated laser photocoagulation versus conventional laser as the primary treatment for symptomatic RAM.

In an earlier study by Kernt M and coauthors, a higher accuracy of navigated focal laser photocoagulation of microaneurysms compared to conventional laser was achieved in patients with diabetic macular edema [15]. However, it is clear that in photocoagulation of macroaneurysms, the same visible end-point as in microaneurysms, treatment can only be achieved with higher laser energy and therefore is associated with increased risk of collateral damage of the retina. From this point of view, the accuracy of photocoagulation has a greater importance specifically in RAM.

In non-symptomatic and non-complicated cases of RAM, observation is considered to be the prefered approach. Hughes EL and coauthors suggested that thrombosis of the RAM confirmed with FA also required only observation since it precluded spontaneous regression of macroaneurysm [4]. Cahuzac A and coauthors, having divided RAM into hemorrhagic and exudative forms, concluded that the exudative form has a better prognosis than hemorrhagic and can be followed up by observation [16]. This also applies to RAM patients with arterial hypertension, compensation of which can lead to regression of the RAM. To exclude self-resolution of the RAM, all patients in our study were observed for 1 month before treatment allowing us to conclude that structural and functional improvement after treatment was associated with laser treatment only. Although an observation period can be extended up to 4 months [8], we chose 1-month observation only to minimize negative consequences of longstanding exudation on functional outcome.

Although the natural course of the RAM can be self-regressive [17] there are several counter-arguments which indicate a demand for the interventional approach. Firstly, non-resolving cases may be complicated by a rupture of the RAM wall and significant preretinal hemorrhage [18] or by BRVO [19, 20], both of which result in significant visual deterioration and require vitrectomy [4]. Secondly, it is worth noting the absence of substantial visual improvement in a significant proportion of patients in our study even after complete resolution of retinal exudation. We believe this could be explained by the prolonged subfoveal detachment accompanied by atrophy of the outer retinal layers in these patients. On OCT examination it was represented by the discontinuation of the ellipsoid zone and outer nuclear layer thinning in the area of resolved subfoveal detachment (data not presented). Similar OCT findings were described in patients with chronic central serous chorioretinopathy [21] and diabetic macular edema patients [22, 23] who demonstrated poor visual acuity following resolution of neuroepithelial detachment and intraretinal cystic fluid. Thirdly, intervention could be recommended if the etiology of the macroaneurysms indicates that spontaneous regression should not be expected. In particular, macroaneurysms associated with macular telangiectasia type 1 appear to have no tendency to be self-resolving [24]. At the same time, both laser photocoagulation and anti-VEGF therapy were effective in these cases [24]. Taking all into consideration, adopting an interventional approach is justified in a significant proportion of RAM cases.

Laser photocoagulation is a generally safe option for RAM treatment. In our study, we did not meet any laser-related complications. In spite of the limited study population, this fact confirms that laser photocoagulation could be the primary option for RAM treatment. However, tissue-sparing approaches and, primarily, anti-VEGF therapy [25] affords a dynamic of decreasing risk for collateral retinal damage. Nevertheless, anti-VEGF therapy is not without its disadvantages since it includes invasive manipulations and typically needs repetitive procedures, as well as not guaranteeing complete resolution of the RAM. Subthreshold laser treatment as an alternative tissue-sparing option demonstrated efficacy similar to conventional laser photocoagulation without attendant adverse effects [8]. However, it is worth noting that although the authors reported no detectable signs of the treatment at the laser application site, nevertheless the calculated total laser energy was quite high (at least of 0.06 vs 0.02 mJ/pulse in our study).

Two general approaches for laser treatment of RAM were described: (1) direct application of laser to RAM surface [3] and (2) indirect treatment of surrounding neuroepithelium [26] (with or without coagulation of feeder and draining vessels [27]). Although direct treatment appears to have a more obvious pathophysiological rationale, this approach may be complicated by disruption of RAM wall in the case of excessive thermal damage [28]. On the one hand, with conventional laser photocoagulation there is no clear boundaries between the direct and indirect approach because the accuracy of the treatment is limited by the human factor (both operator- and patient-associated). On the other hand, the excessive thermal damage may be associated with the risk of subsequent rupture of the RAM wall [28]. In our study, the most important factor determining the total laser energy was the number of laser burns applied. Since the higher than for conventional laser hit rate in treatment microaneurysms was already demonstrated for NAVILAS [13], we concluded that decreased number of laser burns in RAM treatment reflects the higher accuracy of the navigated procedure. Therefore, the navigated approach limits the zone of laser application exclusively to the RAM and, hence, decreases thermal damage and, also, the risk of rupture of the RAM. We believe, that this low-intensity and precise treatment provides the key element of safety of the truly direct RAM photocoagulation.

In conclusion, in this study direct laser photocoagulation as the primary option in the management of symptomatic RAM has demonstrated high efficacy without any laser-associated adverse events. At the same time, this study suggests, that compared to conventional laser photocoagulation the navigated approach has a lower potential risk for collateral retinal damage since it requires application of the lower number of laser burns and less total laser energy during the procedure and, therefore, appears to be in general superior to conventional laser treatment.
